# Retinocytoma associated with bilateral retinoblastoma

**DOI:** 10.4103/0301-4738.60094

**Published:** 2010

**Authors:** Masood Naseripour, Khalil Ghasemi Falavarjani, Siamak Akbarzadeh

**Affiliations:** Iran University Eye Research Center and Eye Department, Rasoul Akram Hospital, Tehran, Iran; 1 Hamedan University of Medical Sciences, Hamedan, Iran

**Keywords:** Bilateral retinoblastoma, retinoblastoma, retinocytoma

## Abstract

A 3-year-old girl presented with left exotropia. Funduscopy demonstrated a retinocytoma associated with five discrete retinoblastomas in the left eye and three discrete retinoblastomas in her right eye. The clinical manifestations and fundus imaging findings are described.

Retinocytoma is a rare retinal tumor that is generally believed to be a benign variant of retinoblastoma.[1–6] The reported proportion of retinocytoma among the population with retinoblastoma has varied from 1.8 to 10%.[1,2,7] The ophthalmoscopic appearance of the retinocytoma, including the presence of a gray translucent mass, intralesional calcification, retinal pigment epithelial alteration and chorioretinal atrophy resembles the spectrum of retinoblastoma regression patterns observed after irradiation.[2]

It is important to recognize retinocytoma clinically and differentiate it from active retinoblastoma as it usually requires close observation rather than active treatment.

Simultaneous occurrence of retinocytoma and retinoblastoma is very rare[1,7,8] and most retinocytomas are stable and demonstrate no tendency to grow or metastasize.[2] We report here a unique presentation of a retinocytoma associated with bilateral retinoblastoma.

## Case Report

**Figure 1:**
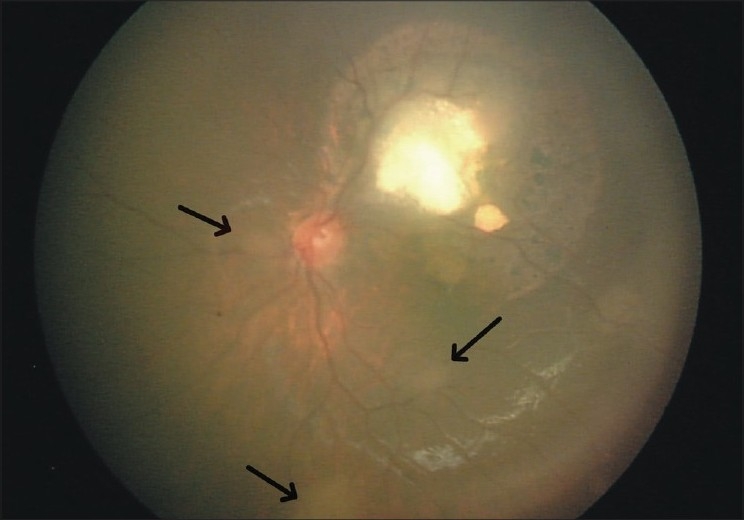
Left fundus photograph showing a gray translucent retinal mass with calcification, retinal pigment epithelial alteration and surrounding chorioretinal atrophy. Three discrete retinoblastomas (arrows) can be seen as translucent tumors at the posterior pole

**Figure 2:**
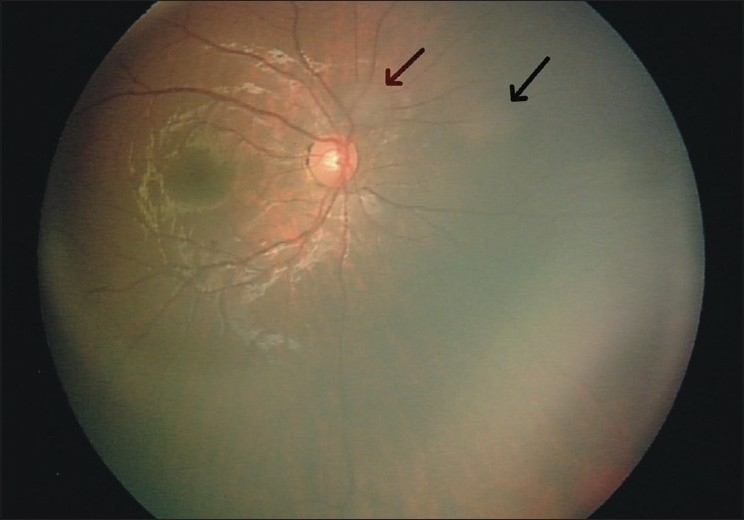
Right fundus photograph showing two foci of retinoblastoma, (arrows) nasal and superonasal to the optic disc

A 3-year-old girl was referred to the oncology service because of left exotropia associated with an intraocular mass. Familial history was not contributory. Examination under anesthesia revealed an elevated semitranslucent mass measuring about 9 × 8 mm in basal dimensions and 3 mm in thickness superior to the left fovea, surrounded by a margin of chorioretinal atrophy and retinal pigment epithelial alteration [Fig. 1]. Some calcification was evident in the central portion of the tumor. This retinal lesion was thought to be a retinocytoma. Five small translucent masses compatible with retinoblastoma were found in the same eye: Four in the posterior pole and the fifth near the ora serrata. Two subretinal seeds were found near the ora serrata as well. In the right eye, two small retinoblastomas were present near the optic disc and another one was found near the ora serrata [Fig. 2]. Anterior segments of both the eyes were normal. No extraocular extension and central nervous system abnormality was found in systemic evaluation and neuroimaging studies. Fundus examination of the parents with scleral indentation was negative for any similar pathology.

**Figure 3:**
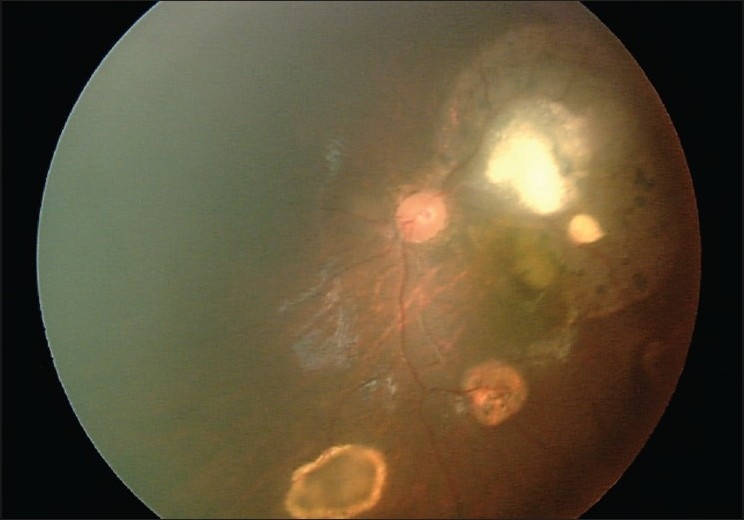
Left fundus photograph showing complete regression of malignant lesions without an obvious change in the size of the retinocytoma lesions

The patient was referred for systemic chemotherapy with a vincristine-etoposide-carboplatin protocol for six cycles. Examinations under anesthesia were performed in between the chemotherapy cycles every 6-8 weeks. In addition, cryotherapy was performed for more peripheral retinoblastoma tumors and posteriorly located malignant tumors were treated using transpupillary thermotherapy. Overall, two sessions of cryotherapy were performed for both eyes as well as three sessions of TTT for the left eye and two sessions for the right eye. At the last examination under anesthesia [Fig. 3], 1 year after starting chemotherapy, her malignant lesions completely regressed in both eyes and no change in the size of retinocytoma was evident in the left eye.

## Discussion

Retinocytoma is considered to be a rare benign phenotypic *RB1* gene mutation and carries similar genetic implications as germinal retinoblastoma.[1,2,5] In familial cases, various members of a family may present with either retinocytoma or retinoblastoma or even a combination of both between the two eyes.[1,2,7] Histologically, retinocytoma is composed of benign appearing cells with numerous fleurettes and lack of necrosis or mitotic activity.[5] Localized deposits of calcium inside the tumor or even in the vitreous may be present.[2,8]

Retinocytoma and retinoblastoma have been reported in different forms and association; in the same family,[1,2,7] in a case with retinoblastoma in one eye and retinocytoma in the fellow eye,[1,7] as two separate foci in the same eye[2,8] and in the parents of a child with retinoblastoma.[1,2,7] Bilateral retinocytoma without any malignant association has also been reported.[9,10] However, based on a search in the Pubmed and Scopus databases from 1915 to 2008, this is the first case of retinocytoma associated with bilateral retinoblastoma.

Although all four diagnostic features of retinocytoma may be present in 10% of the patients,[2] all were documented in our patient [Fig. 1]. Simultaneous occurrence of retinocytoma and retinoblastoma and possible chance of malignant transformation stress the importance of complete examination of both eyes and close follow-up of the patient with a presumed diagnosis of retinocytoma.
